# Insulation Performance and Simulation Analysis of SiO_2_-Aramid Paper under High-Voltage Bushing

**DOI:** 10.3390/nano12050748

**Published:** 2022-02-23

**Authors:** Bowen Liu, Fangcheng Lv, Xiaozhou Fan, Hai Xiao, Hanwen Bi

**Affiliations:** Hebei Provincial Key Laboratory of Power Transmission Equipment Security Defence, North China Electric Power University, Baoding 071003, China; lbw@ncepu.edu.cn (B.L.); xiaohai@ncepu.edu.cn (H.X.); bhw@ncepu.edu.cn (H.B.)

**Keywords:** OIP bushing, nano-modified technology, aramid insulation paper, SiO_2_

## Abstract

The long-term safe and stable operation of oil-impregnated paper (OIP) bushings is of great significance to the operation of power systems. With the growth of OIP bushing, its internal insulation will gradually decay. Aramid insulation paper has excellent thermal aging characteristics and its insulation performance can be improved by using nano-modification technology. In this paper, the nano-SiO_2_ particles were used as the modified additives, and the modified aramid insulation paper was prepared through four steps: ultrasonic stirring, fiber dissociation, paper sample copying and superheated calendering. The microscopic physical morphology and chemical components of the insulation specimens before and after modification were analyzed by atomic force microscopy (AFM), scanning electron microscopy (SEM) and X-ray photoelectron spectroscopy (XPS), and an OIP bushing model based on the modified aramid insulation paper was constructed and its electric field distribution was analyzed. The simulation results show that the use of SiO_2_-modified aramid insulation paper can improve the electric field distribution of OIP bushings and increase the operating life of power transformers.

## 1. Introduction

As an important part of power transformers, oil-impregnated paper (OIP) bushings assume the important roles of leading out the high and low voltage leads of power transformers, fixing the internal lead structure, and achieving insulation between the leads and the transformer tank [1]. Currently, OIP bushings for power transformers usually adopt a structure of multi-layer metal laminations, while the oil-impregnated paper insulation is widely used inside power transformers due to its high thermal stability.

In long-term operation of transformer bushings, due to the combined effect of high temperature and strong electric fields, oil-impregnated paper insulation will be aged with the operation of electrical equipment, thus causing a decline in electrical and mechanical properties. In actual operation, aging transformer oil can be filtered or even changed to remove aging products to restore insulation performance, but insulation paper in the bushing cannot be replaced, so the aging state of the insulation paper determines the aging life of the transformer.

The main components of aramid paper are two polymers, aramid short-cut fiber and aramid pulp [2]. It has the excellent properties of aramid fiber, high strength and high modulus, low deformation, high temperature insulation, corrosion resistance, and is an excellent insulating material due to its light weight, flame retardancy, and abrasion resistance. Aramid insulating paper not only has good electrical properties, but also can operate at 180 °C for a long time, has excellent anti-heat aging properties, and can be used safely and stably for more than 10 years [3]. Therefore, replacing the original cable paper in oil-impregnated paper bushing by using aramid paper was considered to improve the thermal aging resistance characteristics of the oil-impregnated paper insulation. However, the very small radial insulation size of the OIP bushings leads to a large spatial electric field strength, and the dielectric constant of the aramid paper needs to be modified to homogenize electric field distribution inside the bushing. At present, domestic and foreign scholars have made relevant results on the application of nano-modification technology to insulating paper [4,5,6,7,8,9,10,11,12,13,14,15,16,17,18,19,20,21,22,23,24].

In the field of using nanoparticles to improve the electrical properties of traditional insulating paper, scholars have tried to modify insulating paper by adding nano-SiO_2_, nano-Al_2_O_3_, nano-TiO_2_, nano-MgO, and nano-ZnO particles, and the performance of these nanoparticles can be different. The nano-SiO_2_ particles can reduce the dielectric constant of insulating paper, and at a content of 5%, the dielectric constant is 34% lower than that before modification [12]; the thermal conductivity of insulating paper increases with the addition of nano-Al_2_O_3_ compared with the previous one [13,14]; the addition of nano-TiO_2_ increases the frequency breakdown voltage of insulating paper [15]; nano-MgO modified insulating paper has better anti-aging performance [19]; nano-ZnO has better suppressive effect on space charge of insulating paper [19]. Some scholars also used nano-SiC to modify insulating paper for the transformer outlet, and the study showed that field strength in the modified insulating paper was reduced under the action of DC and polarity reversal voltage, and the electric field distribution in the oil-impregnated paper tended to be more uniform [16]. The literature [23,24] shows that nanoparticles with low dielectric constants are more beneficial to improve the breakdown strength of composite nanomaterials, and common nanoparticles for cellulose insulating paper modification are mainly nano-Al_2_O_3_, nano-TiO_2_ and nano-SiO_2_, whose dielectric constants are 9.8, 110 and 4.5, respectively. Meanwhile, scholars proposed a method to prepare low dielectric constant insulating paper using nano-SiO_2_ materials, which also provided ideas for this paper [10].

Therefore, in this paper, nano-SiO_2_ particles were used as the modified additive of aramid insulating paper, and the nano-modified aramid insulating paper was prepared based on nano-modification technology, then the modified aramid insulating paper was tested by atomic force microscopy (AFM), scanning electron microscopy (SEM) and X-ray photoelectron spectroscopy (XPS) to further analyze the effect of nano-SiO_2_ particles on the physical and chemical properties of the aramid insulating paper. Based on the results, an OIP bushing model was developed in Comsol to analyze the effect of modified aramid insulating paper on the actual electric field distribution of power transformers.

## 2. Aramid Sample Preparation and Property Testing

### 2.1. Material Preparation

In this paper, nano-modification technology was used to modify aramid insulating paper. The nano-modified aramid paper samples were prepared using nano-SiO_2_ particles as the modified additives of aramid insulating paper through four steps: ultrasonic stirring, fiber dissociation, paper sample copying and superheated calendering.

Raw materials used in the whole modified aramid sample preparation process include aramid staple fiber, aramid pulp and nano-SiO_2_ particles, among which aramid staple fiber and aramid pulp, as important components of aramid insulating paper, play a decisive role in the material properties of aromatic insulating paper. The selected aramid short-cut fiber is 12 mm in length, about 10 μm in diameter and light yellow in color; the selected aramid pulp is 2 mm in length and light yellow in color; the selected SiO_2_ nanoparticles are about 12 nm in diameter, with a specific surface area of about 200 m^2^/g and are a white solid powder. The aramid short-cut fiber and aramid pulp selected for this paper were purchased from Yantai Teplon Advanced Manufacturing Technology Co. The nano-SiO_2_ powder was purchased from Guangzhou Wanjing Co. (Guangzhou Wanjing Co., Guangzhou, China)

**Figure 1:**
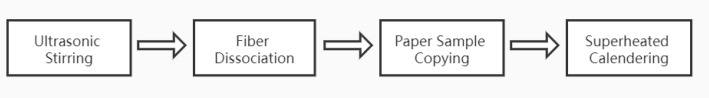
The whole modified aramid insulating paper sample preparation process.

The whole modified aramid insulating paper sample preparation process is shown in Figure 1.

The specific process is as follows:

1. The SiO_2_ nanoparticles with a diameter of about 12 nm are added to deionized water together with a 12% dispersant solution, and the mixed solution is dispersed ultrasonically with the aid of an ultrasonic cleaner;
2. Take a fiber dissociator, add an appropriate amount of aramid short-cut fibers with a length of 12 mm to it, add the previously prepared nano-SiO_2_ particles dispersion solution after being dispersed evenly, and stir it fully for 8 min. Subsequently add an appropriate amount of aramid pulp with a length of 2 mm, and stir it fully for 8 min to obtain a mixed original pulp containing nano-SiO_2_ particles;
3. Use a paper sample copying machine to copy and form the mixed original pulp containing the nano-SiO_2_ particles to obtain dry paper containing the mixture of nano-SiO_2_ particles;
4. The obtained dry paper containing the mixture of nano-SiO_2_ particles is superheated and calendered to obtain an improved aramid insulating paper containing nano-SiO_2_ particles.

### 2.2. Test Methods

In this paper, a Dimension FastScan atomic force microscope (AFM) (Suzhou Feisman Precision Instruments Co., Suzhou, China) and Quanta FEG 250 scanning electron microscope (SEM) ( Carl Zeiss AG, Oberkochen, Germany)were used to test the external physical morphology of modified aramid insulating paper samples containing SiO_2_ nanoparticles to investigate the effect of SiO_2_ nanoparticles on the microscopic morphology of aramid insulating paper. Thermo SCIENTIFIC ESCALAB 250Xi X-ray photoelectron spectroscopy (XPS)(Thermo Fisher Scientific, Waltham, MA, USA) was used to analyze the surface chemistry of the modified aramid insulating paper samples to obtain the composition elements of the modified aramid insulating paper samples.

By controlling the amount of SiO_2_ nanoparticles added during the preparation of the modified aramid insulating paper samples, the optimal modification ratio was investigated, and three groups of modified aramid insulating paper samples with different contents of SiO_2_ nanoparticles were produced. The modified aramid insulating paper samples with 0.5 wt%, 1 wt% and 3 wt% of nano-SiO_2_ particles were labeled as F1, F2 and F3, respectively, and the original aramid insulating paper samples without nano-SiO_2_ particles were labeled as F0.

## 3. Physical Test Results

**Figure 2:**
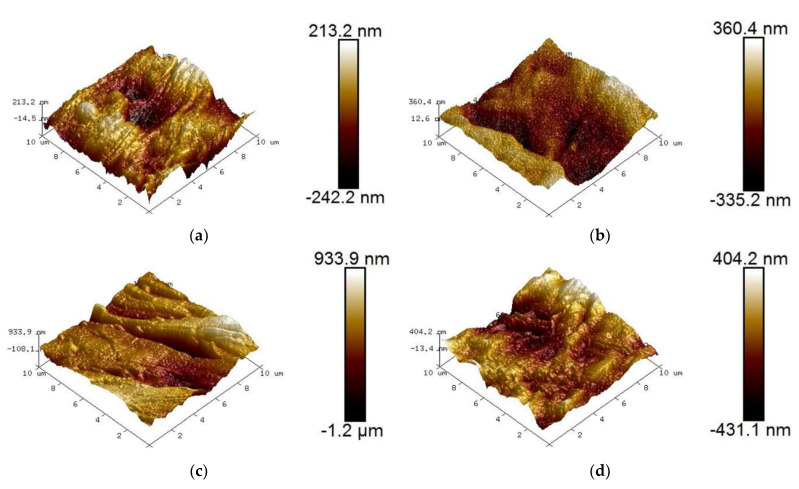
Atomic force microscopy (AFM) test results before and after modification of aramid insulation paper. (**a**) F0 Sample AFM; (**b**) F1 Sample AFM; (**c**) F2 Sample AFM; (**d**) F3 Sample AFM.

The AFM test results of the modified aramid insulating paper samples are shown in Figure 2.

From Figure 2, it can be seen that the surface of the aramid insulating paper samples without nano-modification technology treatment is relatively flat, without obvious concave and convex changes, and overall surface roughness is low. With a gradual increase of nano-SiO_2_ particle filler, grooves on the surface of the modified aramid insulating paper samples increased significantly and surface roughness also increased, but when nano-SiO_2_ particle filler increased to a certain extent, grooves on the surface of the modified aramid insulating paper samples decreased again and surface roughness also decreased.

The results of the surface roughness Rq of the modified aramid paper samples are shown in Table 1. 

**Figure 3:**
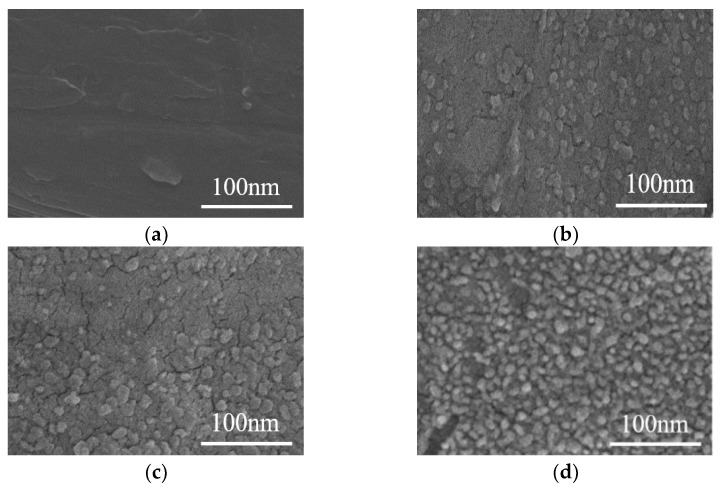
Scanning electron microscopy (SEM) test results (scale bar: 100 nm) before and after the modification of aramid insulation paper (**a**) F0 Sample SEM; (**b**) F1 Sample SEM; (**c**) F2 Sample SEM; (**d**) F3 Sample SEM.

SEM tests were conducted on aramid insulating paper samples treated with the nano-modification technology, and test results are shown in Figure 3. From test results, it can be seen that with a gradual increase of nano-SiO_2_ particle filler, the surface of the aramid insulating paper sample is gradually becoming etched by nano-SiO_2_ particles. The reason is that with an increase of nano-SiO_2_ particle modifier, the degree of etching of nano-SiO_2_ particle modifier on the surface of aramid insulating paper sample gradually increases, the surface of the aramid insulating paper sample is gradually changed, the surface of the substrate on the surface of the sample is gradually peeled off resulting in gradual exposure of the nano-SiO_2_ particle modifier, and the quantity shows an increasing trend.

**Figure 4:**
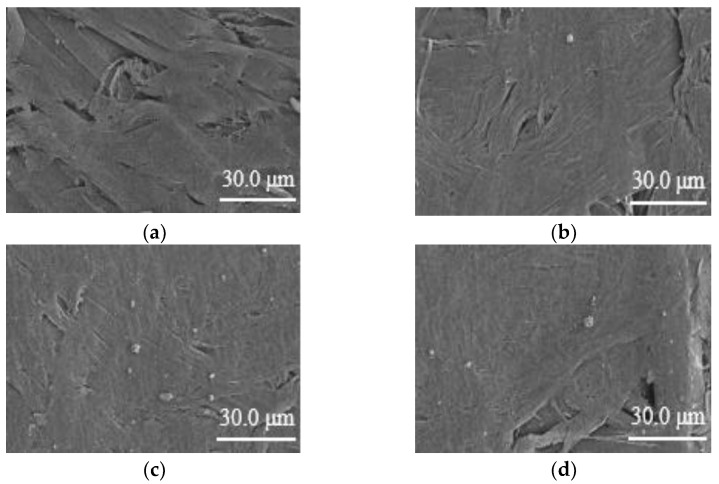
SEM test results (scale bar: 30 μm) before and after the modification of aramid insulation paper (**a**) F0 Sample SEM; (**b**) F1 Sample SEM; (**c**) F2 Sample SEM; (**d**) F3 Sample SEM.

At the same time, as the number of SiO_2_ particle modifiers increased, the surface of the aramid paper sample was not further peeled off by the intermolecular force of the aggregation of SiO_2_ particle modifiers, which led to the reduction of surface roughness. The test results are shown in Figure 4.

## 4. Chemical Test Results

**Figure 5:**
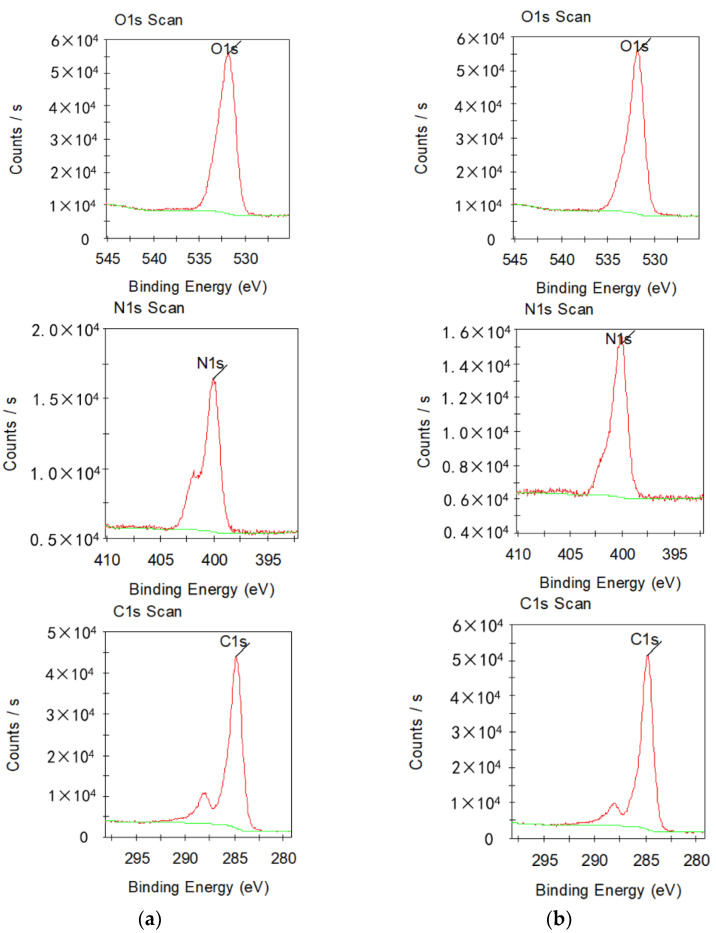
X-ray photoelectron spectroscopy (XPS) test results before and after modification of aramid insulation paper (**a**) F0 Sample XPS; (**b**) F3 Sample XPS.

XPS tests were performed on aramid insulation paper samples treated with nano-modification technology, and the results are shown in Figure 5. Prepared modified aramid insulating paper samples were treated under a high vacuum environment for 6 h, and then were analyzed by XPS scanning on an ESCALABMKII XPS instrument, setting the vacuum level of the analysis chamber used for the experiment to 5 × 10^−6^ Pa, setting the power to 500 W, using C1s as the energy counterpart for the whole XPS test process, and using MgKa rays as the energy source for the XPS test. The results of the whole XPS analysis are shown in Figure 4, where C1s, N1s, and O1s indicate the inner energy levels of C, N, and O.

It can be seen from test results that with a gradual increase of SiO_2_ nanoparticle filler, the characteristic peaks of C1s, N1s and O1s spectra changed, the chemical state of the surface of the aramid insulating paper samples treated by the nano-modification technology changed accordingly, and the contents of C, N and O all increased respectively. The content of the groups on the surface of the aramid fibers changed significantly, with the content of group –C–OH increasing and the content of the group –C=O showing an increasing and then decreasing trend, both of which were beneficial to improving the electrical properties of the aramid insulation paper samples, verifying the effectiveness of this modification method.

## 5. Dielectric Constant Test Results

**Figure 6:**
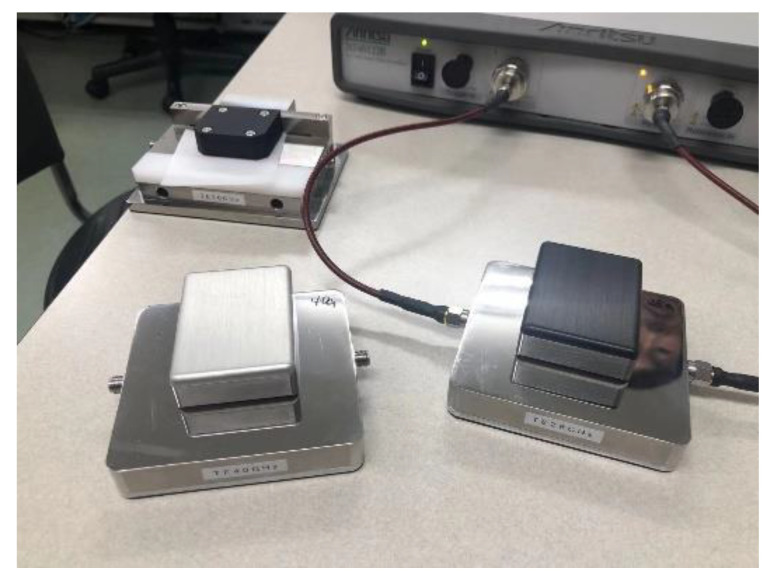
Dielectric constant test platform.

In order to understand the specific aramid modification results, a dielectric constant test was performed on this modified sample, and the test platform is shown in Figure 6. Four modified aramid samples were placed on the platform for testing. All dielectric constant test results are shown in Table 2.

## 6. Finite Element Simulation Model of Transformer Bushings

### 6.1. Structure of the Oil-Filled Paper Condenser Type Bushing

Oil-filled paper condenser type bushings are one of the common types of high-voltage bushings. Although the bushing structure under different voltage levels is slightly different, the structure of the internal capacitor packet is basically the same [7].

**Figure 7:**
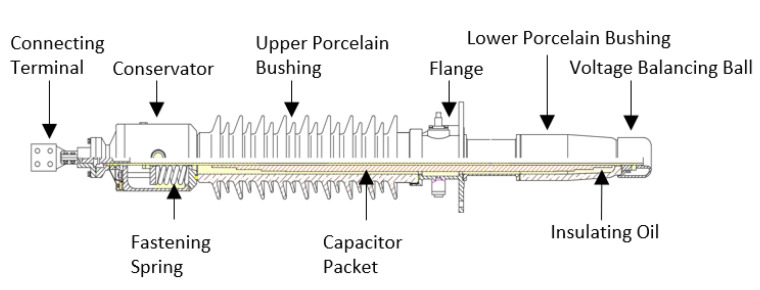
Structure of 72.5 kV bushing.

This paper takes a 72.5 kV OIP bushing as the research object and uses COMSOL multiphysics 5.2a simulation software to simulate the electric field distribution before and after replacement of the capacitor packet. An oil-filled paper condenser type bushing is mainly composed of a connecting terminal, conservator, fastening spring, central guide rod, upper porcelain bushing, grounding flange, flange cylinder, lower porcelain bushing and capacitor packet. The bushing is filled with insulating oil, which together with the capacitor packet composed of insulating paper and aluminum foil forms the oil-impregnated paper insulation system. The length of the bushing is 2245 mm, the length of the upper porcelain bushing is 850 mm, the length of the lower porcelain bushing is 210 mm, the lead-in length of the cable is 1865 mm, the inner diameter of the conductive rod is 35 mm, and the maximum radius of the capacitor packet is 43.6 mm. The structure of this 72.5 kV OIP bushing is shown in Figure 7.

In actual operation, the central guide rod of the bushing carries current and is fixed to the top of the transformer through a grounding flange. The capacitor packet uniformizes the electric field distribution between the central guide rod and the ground potential through an internal insulation system composed of insulating oil, insulating paper and aluminum foil. The thin capacitor packet bears the transformer phase voltage, the electric field strength is large, the operating environment is harsh, and manufacturing process requirements are high. Due to the existence of air bubbles in the capacitor packet, poor impregnation, and air gaps between oil and paper and other process problems, bushing failures and even transformer explosion accidents occur from time to time [25,26,27].

### 6.2. Insulation Principle of Capacitor Packet

The oil-impregnated paper insulation system composed of insulating oil, insulating paper and aluminum foil is the main insulation for the transformer bushing. By alternately winding 0.08 mm–0.12 mm-thick insulating paper and 0.01 mm-thick aluminum foil on the central guide rod to form a plurality of capacitors with the same capacitance value, uniform voltage and electric fields are achieved [28]. Generally, the number of layers of aluminum foil (also known as capacitor plate) in the 72.5 kV OIP bushing is 18, the outermost layer of which is the end screen. The end screen is grounded during operation, which is at the ground potential. The voltage between the center guide rod and the end screen is the sum of the voltages between the plates.

If the error caused by the edge effect of the capacitor plate is ignored, it can be considered the electric flux passing through each level of the plate does not change:

(1) $$DS=2\pi rl\varepsilon_{0}\varepsilon_{r}E_{r}=c_{1}$$

where *D* is the electric flux, *S* is the area of the electrode plate, *ε*_0_ is the dielectric constant in vacuum, *ε_r_* is the relative permittivity of the material, *E_r_* is the radial electric field strength here, *r* is the radius of the electrode, *l* is the length of the electrode, and *c*_1_ is a constant.

**Figure 8:**
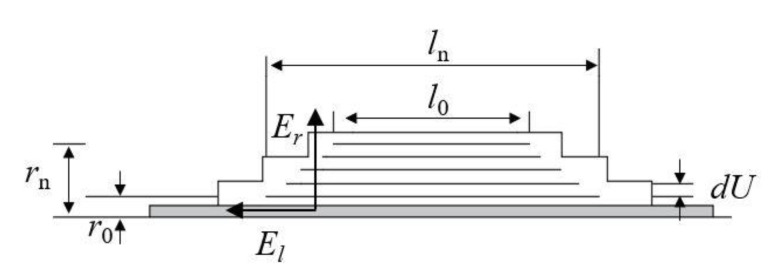
Structure of the bushing capacitor packet.

Structure of the bushing capacitor packet is shown in Figure 8. Suppose the voltage between two adjacent layers of plates is *dU*, and the axial field strength is *E_l_*, there are:

(2) $$dU=-E_{r}dr=E_{l}dl$$

Assuming the axial field strength as a constant, it can be obtained from Equation (1) that:

(3) $$E_{l}=\frac{U}{l_{0}-l_{n}}$$

(4) $$E_{r}=\frac{c_{2}}{rl}$$

(5) $$ldl=c_{3}\frac{1}{r}dr$$

In the formula, *c*_2_ and *c*_3_ are constants. Integrate formula (4) to obtain:

(6) $$\frac{l^{2}}{2}-c_{3}\ln r+c_{4}=0$$

In the formula, *c*_4_ is a constant. Substituting formula (3) and boundary conditions *r = r*_0_, *l = l*_0_*; r = r_n_*, *l = l_n_* into formula (5), it can be solved as follows:

(7) $$E_{r}=\frac{l_{0}+l_{n}}{\ln(r_{n}/r_{0})}\cdot \frac{U}{2rl}$$

From formula (7), it can be seen when *E_l_* is a constant, the product of *E_r_* and *rl* is inversely proportional. The radial field strength of the capacitor packet presents a U-shaped distribution, the electric field strength is higher near the end screen and the center guide rod, and the electric field strength in the middle part is lower. Through the equal-capacitance design method, the length of each plate in the packet is solved by using the fact that the capacitance of each plate is equal. Suppose the interlayer capacitance is *C_k_*, then:

(8) $$C_{k}=\frac{2\pi \varepsilon_{0}\varepsilon_{r}l_{1}}{\ln(r_{1}/r_{0})}=\frac{2\pi \varepsilon_{0}\varepsilon_{r}l_{2}}{\ln(r_{2}/r_{1})}=\cdots =\frac{2\pi \varepsilon_{0}\varepsilon_{r}l_{n}}{\ln(r_{n}/r_{n-1})}$$

It can be seen from formulas (7) and (8) that replacing the insulating material of the capacitor packet causes a change in the dielectric constant, resulting in a change in the electric field strength.

### 6.3. Finite Element Simulation Theory

The finite element method is a numerical calculation method based on the difference method and the variational principle. It was used in the structural mechanics in early days and was then gradually applied in the fields of electromagnetic field and fluid mechanics [8].

The electric field of the high-voltage bushing is generated by the industrial-frequency alternating current passing through the center guide rod. The frequency is 50 Hz, which can be reduced to a two-dimensional axisymmetric electrostatic field problem. The Laplace equation of the potential is:

(9) $$\nabla^{2}\varphi =\frac{\partial^{2}\varphi }{\partial x^{2}}+\frac{\partial^{2}\varphi }{\partial y^{2}}+\frac{\partial^{2}\varphi }{\partial z^{2}}=0$$

The boundary conditions are the first and second types of boundary conditions, which are:

(10) $$\varphi =f_{1}\left(p\right),\frac{\partial \varphi }{\partial n}=f_{2}(p)$$

Solving the electric field of the transformer bushing in the COMSOL finite element software can be divided into six steps, including selecting the electric field module according to the physical field of the problem to be solved; building the model according to the structure; setting the material parameters of each part; setting the load and boundary conditions; performing meshing according to the characteristics of the electric field and selecting a solver to solve the model [29].

### 6.4. Parameters and Settings of Finite Element Simulation

**Figure 9:**
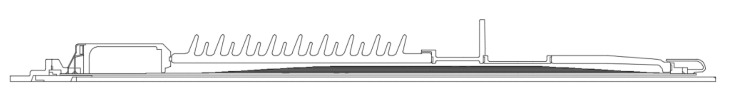
Finite element model of 72.5 kV transformer bushing.

Due to highly axisymmetric characteristics of the transformer bushing, the three-dimensional model can be simplified into a two-dimensional axisymmetric model for calculation, and this has little effect on calculation accuracy. According to the structure of the transformer bushing, a two-dimensional symmetrical model is established in COMSOL, as shown in Figure 9.

We add material properties to the solution domain according to bushing structure. In the simulation calculation, the relative permittivity of each material is set as shown in Table 3:

According to actual working conditions of the transformer bushing, a working voltage of 41.9 kV is applied to one end of the center guide rod, and a working current of 630 A is applied to the other end. The pole plate in the capacitor packet is set to a floating potential, and the end screen is connected to the flange by a grounding lead, which is set to the ground potential in the model, and the flange is also set to the ground potential. An air area at the length of 1.5 m and a fuel tank area at the length of 0.5 m are set outside the bushing to simulate actual working conditions.

Since the thickness of the aluminum foil is only 0.01 mm, the mesh size will be extremely small, the number of meshes will be greatly increased, the quality of the mesh will be low, and the calculation accuracy will be affected when the finite element method is used for meshing. Setting it as a single-layer material in the simulation can avoid meshing problems.

## 7. Analysis of the Simulation Results

**Figure 10:**
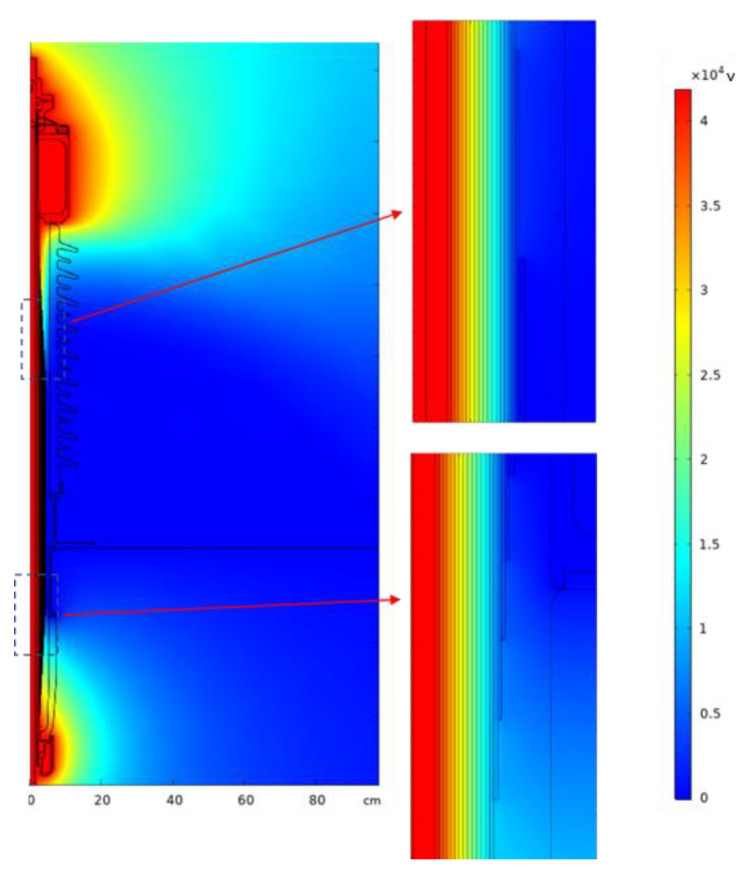
Electric field distribution of the transformer bushing.

**Figure 11:**
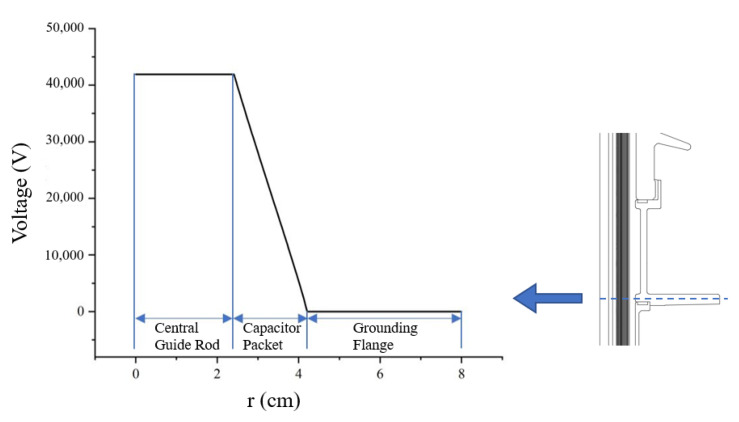
Voltage distribution of the transformer bushing.

The simulation yields a cloud diagram of electric field distribution of the transformer bushing as shown in Figure 10. A cut-off line is taken at the middle of the bushing capacitor packet (near the flange) and the voltage distribution along the line is obtained as shown in Figure 11.

**Figure 12:**
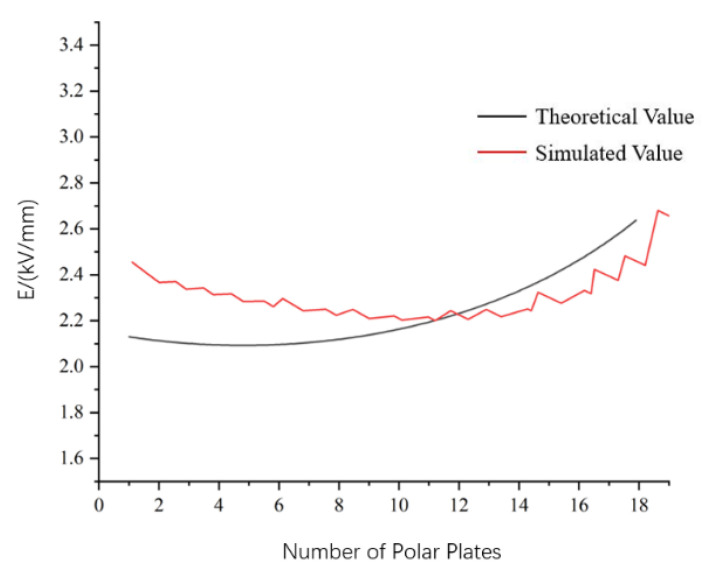
Difference of the simulated value and the theoretical value.

The radial electric field distribution of 72.5 kV bushing can be calculated using Equation (7). As shown in Figure 12, it can be seen that when the bushing is operated at the rated phase voltage, the electric field intensity in the bushing capacitor packet shows a “U”-shaped distribution, with the highest electric field intensity at the outermost end screen of the capacitor packet and the lowest field intensity at the middle part. At the same time, Figure 12 also shows the simulation of the electric field intensity; you can see the simulation value and the theoretical value are quite close. The maximum value of the electric field intensity through simulation is 2.68 kV/mm, and the electric field intensity distribution of the capacitor packet is quite uniform, which is also a curve showing a “U” type distribution, the same as the theoretical value. The difference between the simulated value and the theoretical value at the innermost side of the capacitor packet is large, probably due to the large difference between the parameters of the metal guide rod and the insulation paper, resulting in a large calculation error.

**Figure 13:**
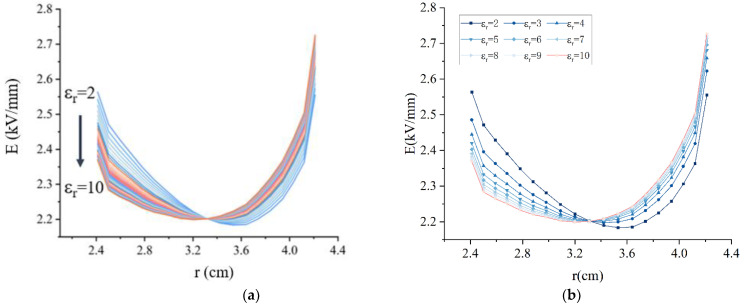
Electric field intensity distribution of the capacitor packet. (**a**) Parametric scanning results; (**b**) Comparison of electric field intensity at different position.

To investigate the effect of the relative permittivity of the capacitor packet on the electric field intensity of the OIP bushing, a parametric scan is performed using COMSOL simulation software. A variety of electric field distributions were obtained by setting different dielectric constants. By comparing each electric field distribution, the dielectric constant for optimal electric field intensity distribution can be found. Set the dielectric constant variation interval as (2,10) and the variation step as 0.2. The simulation solves the electric field intensity at each value, and takes the electric field intensity of the same position of the capacitor packet for comparison, as shown in Figure 13. From Figure 13, it can be seen the electric field intensity exhibits a rotation around a point in the center as the dielectric constant changes. In the inner part of the capacitor packet, the electric field intensity shows a decrease with increasing dielectric constant; near the outer part of the capacitor packet, the electric field intensity increases with increasing dielectric constant.

**Figure 14:**
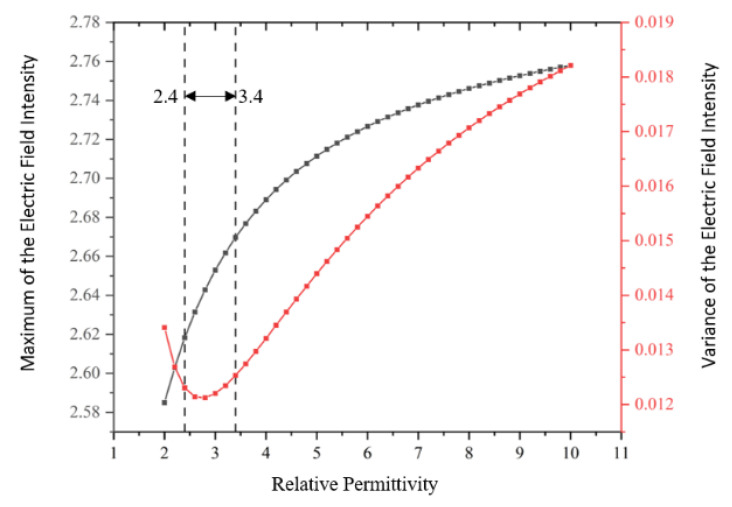
Maximum and variance of the electric field intensity.

The maximum and variance of the electric field intensity in the capacitor packet under each electric field distribution are calculated, as shown in Figure 14. High local electric field intensity will cause partial discharge, which will lead to insulation degradation and even insulation breakdown, so maximum electric field intensity in the capacitor packet needs to be controlled. The capacitor packet electric field intensity variance is used to characterize uniformity of the electric field intensity inside the capacitor packet, and a uniform electric field environment is beneficial to the long-term work of the insulation material.

As shown in Figure 14, when relative permittivity is small, the electric field is uniform and the maximum electric field intensity is small, and where the relative permittivity is 2.8, the electric field intensity variance is smallest and the electric field is most uniformly distributed. However, fabrication of the material does not guarantee exact control of the dielectric constant to 2.8. Controlling the relative dielectric constant between 2.4 and 3.4 can achieve a more uniform electric field and a smaller maximum electric field intensity.

## 8. Conclusions

In this paper, nano-modification technology was used to modify aramid insulation paper, and the physical and chemical properties of the aramid insulation paper before and after modification were tested and analyzed. Based on the technical parameters of the modified aramid insulation paper, a model of OIP bushing was constructed, and its effect on the electric field distribution was studied. The main conclusions of this paper are as follows:

1. The surface of aramid insulation paper sample can be effectively stripped by nano-modification technology, which increases the degree of etching of a nano-SiO_2_ particle modifier on its surface gradually and the surface roughness of the sample, making the surface gouge situation increase significantly. However, with the increase of nano-SiO_2_ particle modifier content, it is influenced by the aggregation of nano-particles into clusters, which leads to the reduction of its surface roughness instead.
2. After comparing the samples of aramid insulation paper modified with nano-SiO_2_ particles with those of aramid insulating paper without nano-SiO_2_ particles, it can be seen that with the gradual increase of nano-SiO_2_ particles filler, the chemical state of its surface changed accordingly, the content of groups on the surface of aramid fiber changed significantly, the content of group-C-OH increased, and the content of group-C=O showed a trend of first increasing and then decreasing.
3. Electric intensity has the smallest variance and the electric field distribution is the most uniform. However, the manufacturing process of the material does not guarantee that the dielectric constant is accurately controlled at 2.8. Controlling the relative dielectric constant between 2.4 and 3.4 can achieve a more uniform electric field and a smaller maximum intensity. In this paper, the modified aramid paper insulation dielectric constant is basically close to the range of 2.4–3.4, which can achieve a more uniform electric field and a smaller maximum electric intensity.

## Figures and Tables

**Table 1:** Modified sample surface roughness.

Sample Number	Rq/nm
F0	88
F1	134
F2	480
F3	229

**Table 2:** Dielectric constant of modified sample.

Sample Number	Dielectric Constant
F0	2.06
F1	2.13
F2	2.65
F3	2.16

**Table 3:** Relative permittivity of each material.

Material	Insulating Oil	Oil-Impregnated Paper	Oil-Impregnated Aramid Paper	Porcelain Bushing	Air
*ε_r_*	2.2	3.8	4.9	7	1

## Data Availability

The data presented in this study are available in Insulation Performance and Simulation Analysis of Nano-modified Aramid Applied in High-Voltage Bushing.
